# Determination of the CO-_2_ Tension of the Alveolar Air and the H-ion Concentration of the Blood and Urine

**Published:** 1917-03

**Authors:** La Rue Colgrove

**Affiliations:** Elmira, N. Y.


### BUFFALO MEDICAL JOURNAL

Yearly Volume 72 MARCH, 1917 Number 8

### ORIGINAL ARTICLES

The right is reserved to decline papers not dealing with practical medical and surgical subjects, and such as might offend or fail to interest readers. Contributors are solely responsible for opinions, methods of expression and revision of proof.

### Determination of the $CO_2$ Tension of the Alveolar Air and the H-ion Concentration of the Blood and Urine.

By LA RUE COLGROVE, M. D., Elmira, N. Y.

The methods are frequently the only means available to the physician for acquiring information of vital importance giving him an accurate picture of the reaction of the blood, which is so essential in those pathological conditions which are clinally recognized as acidosis or acid intoxication. Recent studies have revealed the fact that acidosis, apart from diabetes, may exist in many conditions previously unsuspected.

Henderson and Palmer in their studies on acidosis suggest that acidosis may be assumed to exist when there is no diminution in the acidity of the urine after the administration of an alkali equivalent to a litre of a deci-normal solution. In other words the urine of a normal person should become alkaline after the ingestion of 8 grams of Bicarbonate of Soda. This also serves as a guide to the indication for alkali therapy. If the urine shows a diminished acidity after the ingestion of 4 grams of Bicarbonate of Soda alkalies are not indicated.

Many unexplained and sudden deaths have occurred especially following operations in which neither the disease nor the operation itself were held responsible, and such factors as hemorrhage, shock, infection, pneumonia, having been excluded, may have been due to acidosis. What is acidosis? Physiology teaches us that the normal reaction of the blood is slightly on the alkaline side of neutrality. It is very constant in its reaction varying but slightly in health or disease and any variation in its reaction in disease is always toward acidity. Any appreciable change toward that end is incompatible with life and death speedily ensues.

We remember from our Physico-Chemistry that the reaction of any fluid depended upon the amount of potential H-ions and Oh-ions which it contains. Pure water being used as the base of neutrality contains equal numbers of H and Oh-ions. Hence a solution of acid reaction must contain more H-ions than water and be in excess of the Oh-ions.

The acidity of a solution or the numbers of H-ions which it contains is spoken of as the H-ion concentration. The H-ion concentration of the blood is very constant varying within narrow margin in health or disease. When it is increased, or what is the same thing if the alkali reserve is diminished, we have then a condition known as acidosis.

What then is the normal H-ion concentration of the blood and what means have we of determining it? A normal acid is one having a gram of potential H to a liter of water.

Diluting a normal acid 10 times we have naturally a decinormal acid, continuing the dilution 7 decimal points, we have a solution containing 1-10,000,000 grams of H to a liter which is identical to the amount contained in water which as we know is the base for neutrality. Instead of writing the large number the log arithmic notation of the number is used leaving out the minus sign as suggested by Sörenses. Hence the H-ion concentration of water is Ph-7.

It will be observed the smaller the number the greater the acidity, and as the normal reaction of the blood is on the alkaline side of neutrality its H-ion concentration varies between Ph-7 and Ph-8. Its neutral point 7 being reached only in severe acidosis and Ph-8 after giving an unusual amount of alkalies.

Now while the means for determining it are simple, they are beset with difficulties which render their application at the bed side rather impractical.

We have, however, an indirect means of ascertaining the acidity of the blood, which is extremely simple, accurate, requiring only 2 or 3 minutes time, with no discomfort to the patient and readily carried out at the bed side.

"The Determination of the Co-2 tension of the Alveolar air." By its determination we can judge the degree of acidosis present, it enables us to foresee an impending coma and gives us accurate information as to indication of alkali therapy.

The mechanism by which this slightly alkaline reaction of the blood, so essential for the maintenance of life, is maintained, is one of the most wonderful provisions of nature.

Just consider that every metabolic process of the body, pours into the blood stream acid by-products. Crile and others have shown that every action of the body, emotion, fright, exercise, infection, anaesthesia, surgical operation, trauma results in the formation of acid by-products, in fact the process of life itself is an acid forming one. A steady pouring of acid by-products into the circulation. What is the mechanism by which this excessive acidity is neutralized and the alkaline reserve of the blood maintained at such constancy? The explanation of this is, first, in the composition of the blood itself. The alkali reserve of the blood is due to Bicarbonate of Soda, mono-soda phosphate di soda phosphate and proteids. A solution containing these chemicals will retain its same alkalinity after the addition of considerable acids, although the balance between the two salts will be altered. The chief provision for the acid-base equilibrium is the excretory function of the kidney and lung. The non-volatile acids combine with the base in the blood and are excreted by the kidney as harmless salts, while the volatile acid as Co-2 is excreted by the lungs. If owing to some disturbance in metabolism acids are produced faster than they are excreted or if the excretory organs are impaired, the alkali reserve of the blood being limited, there will naturally result a disturbance of the normal acid-base equilibrium, with the resulting acidosis. Here we see another of natures phenomena. The action of the kidney in separating an acid urine from an alkaline blood, leaving the base for further acid neutralization.

Recent studies on the physiology of respiration have shown that the stimulation was not due to the lack of O in the tissues as believed by the old physiologist, nor as maintained as recently as 1905 by Haldane and Priestly, that the specific stimulation of the respiratory center in the medulla was due to Co-2. This conception has been recently broadened by two Danish Physiologists, Wiesterstein and Hasselbalch, that Co-2 stimulates the respiratory center simply because it is an acid, that any other acid would have the same effect. Respiration then depends upon the H-ion concentration of the blood, as the reaction leans toward acidity, we have an increase stimulation of the respiratory center as evidenced by an increased pulmonary ventilation frequency in respiration and pulse dilatation of the nostrils, etc. Efforts on the part of the organism to maintain the normal reaction of the blood, by lowering its Co-2 tension compensatory for the increase non-volatile acids, which leaves the blood by a slower route, via kidneys. This explains the increased respiration pulse and thirst after violent exercise. As all muscular action results in the formation of acid by-products. Hence we see that respiration and reaction of the blood are interdependent.

The measure of the Co-2 tension of the Alveolar air, which is identical to that of the blood is an index to the degree of acidosis. As the non-volatile acids increases the Co-2 tension diminishes and as acidosis is due to the presence of non-volatile acids the measurement of the amount of Co-2 in the blood or Alveolar air gives us the degree of acidosis. The variation of the Co-2 tension of the blood, is an attempt on the part of the organism to maintain the normal constant reaction of the blood.

Crile has shown that anesthesia is an acid forming process and that the stage of unconsciousness is due to increased acidity. In nitrous Oxide anaesthesia the acidity is more rapid and as we know anaesthesia is more quickly obtained.

During my recent service at the A. O. Hospital all patients coming to the operating table, other than emergency cases, were given preliminary treatment of Bicarbonate of Soda, fruits especially the Citrus fruits and forced Carbohydrate feeding. Instead of the usual enema the morning of the operation, an enema containing 1 oz. of Bicarbonate of Soda to a quart of water was given. It not only cleansed but some of the soda was retained and absorbed. The urine was invariably alkaline at the beginning of the anaesthesia. In every instance immediately after the operation its reaction would be acid, showing that anaesthesia, surgical attack and nervous strain are acid producing factors.

If this simple preparatory treatment of Carbohydrate feeding and alkali therapy is given prospective surgical patients, many of the annoying post-operative symptoms may be avoided, as vomiting, gas, pain, distension, etc., all an expression of an acid intoxication. Experience has taught us that patients suffering from Ca or ulcer of the stomach, or any condition in which the nutrition is poor, are bad surgical risk. The lessened food intake has already produced a diminished alkali reserve and adding to this the increase acidity resulting from the operation, we may have a H-ion concentration so high that the neutralizing mechanism of the organism is unable to re-establish the normal alkalinity of the blood and death results from acidosis. The post operative treatment is no less important. An early attempt should be made to give alkalies and sugar. The early feeding of gruel, orange and grape-fruit juices, fruit ices are well borne and shorten the hour of pain, vomiting, etc. Morphine given before the operation does not add to the acidosis, but it should be withheld after the operation if possible as it inhibits the neutralizing mechanism.

Many bad surgical risks can be made a fairly good one by increasing the alkali reserve of the body before operating by carbohydrate feeding and alkali therapy.

That acid intoxication plays an important role in infectious diseases there can be no doubt. In these cases the patients are too sick to eat, an inadequate food intake or the food may be too low in carbohydrate. Hence the rational high caloric feeding in such protracted diseases as typhoid fever.

Fishier in his new work on Oedema and Nephritis has shown the intimate connection between acidosis and Odema. He explains the reason why alkalies have always been so beneficial in this condition, maintaining that the water in our bodies is held in combination with colloids, analogous by the absorption of water by gelatine which is greatly increased by the addition of a little acid. In Oedema the increased retained acids have caused the tissues to absorb more water.

As the swelling of gelatine can be reduced by adding alkalies or salts so is Oedema reduced by the giving of alkalies and Sulphate of Magnesia.

In pregnancy the symptoms of acid intoxication varies from a mere morning sickness to those alarming ones of pernicious vomiting, headache, convulsions and coma. That this is an acid intoxication is evidenced by the excretion of a highly acid urine with much ammonia and lowered Co-2 tension of the Alveolar air. The diminished alkali reserve as a result of nausea and vomiting added to the acid intoxication resulting from the pregnancy itself, gives us an indication for vigorous alkali treatment with carbohydrate feeding. If the stomach is unable to retain such treatment, they should be given per rectum or intervenously. As prevention is the watch word in medicine, serious complication in pregnancy may be prevented by giving alkalies sufficient to maintain a neutral reaction of the urine to methyl red.

We all know from practical experience that in certain diseases as diabetes, nephritis, acute inflammatory rheumatism that a large amount of alkali is necessary to render the urine alkaline and that the patients are always relieved by its administration. This increase ''Alkali tolerance'' as Sellards terms it is due to the fact that owing to the acidosis present, with the consequent depletion of Sodium Bicarbonate in the tissue, a large amount of the Alkali must first be used to restore the depleted cells before it neutralizes the acid in the blood.

In diabetes, the disease in which acidosis plays the most important role, we have as the result of faulty metabolism an incomplete oxidation of fats into acetone bodies as aceto acetic acid acetone beta oxybutyric acid, etc.

The patient a victim of acid intoxication an increase of non-volatile acids in the blood. While the examination of the urine informs us as to the kind and amount of acids excreted from the body, it gives us no information as to the retained acids within the body which is the most important to know.

The Ferric Chloride test even in approaching diabetic coma may be very weak and on the other hand when the patient is comparatively normal, its reaction may be very strong making it an unreliable test as to the patient's real condition.

We have at our command, however, by the "Determination of the Co-2 tension of the Alveolar air" a method which gives us an accurate picture of the condition which prevails in the blood and enables us to judge the severity and course of the disease. A constantly falling Co-2 tension is a serious warning and of great prognostic value.

Poulton who has given considerable study to the prognostic value of the Co-2 tension, says that values between 30 and 35 M M may be taken as representing considerable acidosis and demanding treatment but there is no immediate dangers to be feared. Values between 30 and 20 are, however, of much more significance and indicate that the patient is nearing the border line. If the tension falls to 20 or lower the patient has reached a point in which coma may be expected at any moment and requires drastic treatment. Observations made at the Peter Bent Brigham Hospital on patients in diabetic coma showed that the Co-2 tension varied between 6 and 10 M. M., which means that the expired air contained only about 1% of Co-2 as compared to 5% normal and 40 M. M. pressure.

The easiest method of determining the Co-2 tension of the Alveolar air is the one suggested by Plesch as modified by Higgins. It comprises first the collection of the expired air and secondly its analysis.

It is based upon the principle that if a patient breathes into and out of a closed space as a rubber bag holding approximately 1000 C. C. for a period of time, not exceeding the time required for one complete cycle of the circulation or 40 seconds, that the diffusion of gases will be the same as the air in the lungs, which represents the same Co-2 tension as the blood. The normal tension with the patient at rest varies from 40 to 45 M. M.

The respirations must not be too rapid or shallow otherwise we would have too low an estimate. If continued longer than the time required for one cycle of the circulation it would give us an estimate too high. Experience has shown that if one will take 4 or 5 deep and slow respirations that the result obtained will be uniformly accurate, varying not more than 2 or 3 M. M. A variance which for clinical purposes may be disregarded.

The analysis of the collected air is extremely simple and depends upon the fact, that if the air from the bag is passed through a solution of bicarbonate of soda containing an indicator until the solution is saturated, or until no further change or color takes place the reaction of that solution will depend upon the relative amount of Bicarbonate of Soda or Co-2 present. The color thus obtained is matched with a standard phosphate solution containing the same indicator. A certain color indicating a certain reaction.

These Standard Phosphate tubes are prepared in the following manner, mixtures of Co-2 of varying percentages were analyzed by the usual gas analysis apparatus and the percentage of Co-2 found multiplied by the barometric pressure gives the Co-2 tension in M. M. These different gas mixtures of known per cent were then blown through a bicarbonate indicator solution and the resultant color matched by phosphate mixtures containing the same indicator. These solutions are so prepared as to give accurate results when the determination is carried out at a temperature between 68 and 77 F.

In conclusion, the determination of the Co-2 tension of Alveolar air gives us a simple clinical test for the detection of acidosis, a condition which exists in a great variety of pathological cases.

Secondly: It has great prognostic value, enabling us to foresee and perhaps forestall an impending coma.

And thirdly: It gives us a rational use and control of alkali therapy.

---

**Syphilis Inherited From Paternal Great Grandfather.** Gaucher, Bull. de l'Acad. de Med., Sept. 26, 1916, reports three infants, one backward, almost idiotic; one with projecting median superior incisors, deep palatine vault, scoliotic, and with adenoids; a third with entero-colitis and adenoids. All three were breast-fed. The serum reaction was positive in the children and the father, negative in the mother. The father was a virgin when married (note that the French language has a masculine word for this meaning), is now 39 years old and presents no sign of acquired syphilis. His father dubious (or the word may have the literal meaning of squint-eyed), no acquired syphilis, dead of cerebral haemorrhage. Great grandfather died young, paraplegic (syphilitic).

